# Antioxidant Biocomposite Films Based on Grape Stalk Lignocellulosic Fractions and Biodegradable Polyesters

**DOI:** 10.3390/polym17111525

**Published:** 2025-05-29

**Authors:** Irene Maté, Lorena Atarés, Maria Vargas, Amparo Chiralt

**Affiliations:** Instituto de Ingeniería de Alimentos-FoodUPV, Universitat Politècnica de València, 46022 Valencia, Spainmavarco@tal.upv.es (M.V.)

**Keywords:** PHBV, PBS, grape stalk, antioxidant capacity, thermal stability

## Abstract

Grape stalk (GS) from winemaking is a waste rich in antioxidant compounds that can be valorized to obtain active food packaging materials. Biocomposite films of poly (butylene succinate) (PBS) and poly(3-hydroxybutyrate)-co-hydroxyvalerate (PHBV) with 10% of GS particles, previously submitted or not to subcritical water extraction at 170 °C and 180 °C, were obtained by melt blending and characterized. The fibres were better integrated in the PHBV matrix than in PBS, while other molecular compounds from the fillers were released to the polymer matrix, allowing for their antioxidant action. Fillers promoted the stiffness of PBS films (11–44%), reducing their resistance to break and extensibility by 25%, without significant changes in polymer crystallinity or thermal stability. However, this reduced the crystallinity (13%) and thermal stability of PHBV films, decreasing their rigidity (55%). All fibres promoted the oxygen barrier capacity in composites (by about 20–35% for PBS and PHBV, respectively) while also providing them with UV light blocking effects. This barrier effect enhanced the ability of the films to preserve sunflower oil against oxidation, while in PHBV composites, the migration of antioxidant compounds was also detected. No remarkable differences in the effects of the different GS fillers on the properties of composites were detected.

## 1. Introduction

Biocomposites made from biopolymers and natural lignocellulosic fibres from agricultural waste and other plant fibres have attracted significant interest due to their sustainability [1] and biodegradability, reducing long-term environmental impact [2]. The use of lignocellulosic materials as fillers in the biopolymer matrix can provide biocomposites with modulated properties, making them the most cost-effective since lignocellulosic waste is generally low-cost and abundant [2]. A variety of biopolymers are being studied for composite production, among which the biodegradable polyesters poly (butylene succinate) (PBS) and poly(3-hydroxybutyrate)-cohydroxyvalerate (PHBV) are promising alternatives to conventional non-biodegradable plastics.

PBS is an aliphatic, potentially bio-based polyester that is considered one of the most interesting compostable polymers because of its good combination of mechanical strength, ductility, toughness, and impact resistance [3]. PBS is chemically synthesized by the polycondensation of 1,4-butanediol and succinic acid [4]. PBS also shows good melt processability with a remarkable thermal resistance and heat deflection temperature (HDT) of over 90 °C and biodegradability when exposed to compositing conditions [4,5]. Its demand has been rising in recent years [6], even though it is more expensive than conventionally used non-biodegradable petroleum-based polymers [5]. Nevertheless, due to its limited Young’s modulus and its susceptibility to sudden degradation during melt processing at high temperature, PBS is often blended or reinforced with other polymers or fillers to improve its processability, stiffness, and mechanical strength and its limited barrier properties [7,8,9].

PHBV is a copolymer of the polyhydroxyalkanoate family produced by microbial fermentation, which is biosynthesized by bacteria as a carbon and energy reserve [10,11]. PHBV shows moderate thermal stability and good tensile strength and gas barrier properties, making it suitable for food packaging. However, PHBV has some limitations in high-temperature applications [12] and softens too much at moderate temperatures, which is a challenge during the heat sealing and thermoforming processes.

Different fillers, such as natural fibres from lignocellulosic materials, have been added to PBS and PHBV materials for their reinforcement and to reduce the production cost of these materials while enhancing compostability [5,6]. Nevertheless, the incorporation of such fillers promoted significant changes in the physical properties of the polymer matrices that may affect their functionality [4]. In this sense, fully biodegradable composites of PHBV and up to 30% lignocellulosic fibres from almond shell and Oryzite^®^ were developed with no significant reinforcing effect, but an improved processing window compared to pure PHBV was observed [13]. Increasing amounts of vine shoot particles were incorporated into a PHBV matrix by melt extrusion, reducing the tensile and water barrier properties and thermal stability of the films [14]. PBS biocomposites with cellulose fibres from rice straw showed reinforced mechanical properties and an improved oxygen barrier compared to pure PBS films [15]. The mechanical performance of PBS films was also improved by incorporating grape stalk particles as a filler material [16].

Grape stalk (GS) is a low-value waste from the wine industry, which represents 3–5 wt% of processed grapes [17]. Wine production consumes 34.1 Mt of grapes worldwide, with Italy, France, and Spain being the main producers in Europe [18]. GS is a lignocellulosic material with 17–26% lignin, 20–30% cellulose, 15–20% hemicellulose 6–9%, and 16% of tannins, which represents 80% of GS phenolic compounds [19,20,21]. Phenolic richness provides GS residue with antioxidant activity, which could be exploited when developing antioxidant materials for food packaging to extend the shelf-life of oxidation-prone foods. Maté et al. [22] obtained water-soluble and -insoluble antioxidant fractions from GS, with high phenolic richness, by applying subcritical water extraction (SWE) at 170 °C and 180 °C. These fractions could be used for different applications, such as the development of active packaging. The use of SWE represented a green method for fractionating lignocellulosic biomass, producing phenolic-rich extracts with non-cellulosic compounds and insoluble fractions enriched in cellulose [23]. This fractionation is caused by the high extractive power of water under subcritical conditions, when its properties selectively change with temperature, improving the solubility of less polar compounds to the level achieved with some organic solvents [24,25].

Both GS powder and its fractions enriched in cellulose by SWE could be used as fillers to obtain biocomposites useful for the antioxidant packaging of foods, with reduced cost and tailored properties. The potential use of grape stalk or its cellulose-enriched fractions as antioxidant fillers in biodegradable food packaging materials has not been previously studied.

In the present study, GS powder and the cellulose-enriched lignocellulosic fractions obtained by using SWE at 170 and 180 °C were incorporated as fillers into PBS and PHBV matrices, aiming to characterize the composite properties in terms of microstructure, polymer crystallization, thermal stability, mechanical and barrier yield, and optical properties. Likewise, the antioxidant capacity of the biocomposite films was analyzed to validate their performance in preserving foods from rancidity, through their capacity to prevent sunflower oil oxidation.

## 2. Materials and Methods

### 2.1. Materials

The poly (butylene succinate) (PBS) biopolyester in the form of pellets was supplied by Mitsubishi Chemical Corporation (Tokyo, Japan) as BioPBS FZ91PM. Poly(3-hydroxybutyrate-co-3-hydroxyvalerate (PHBV) was obtained from Enmat Y1000P, with a hydroxyvalerate fraction of 2% mol. Phosphorus pentoxide was supplied by VWR Chemicals (Leuven, Belgium) and magnesium nitrate by Panreac Química (Barcelona, Spain). Grape stalks of the Bobal variety (origin Requena, Spain) were obtained from the winemaking process performed in the UPV pilot-plant winery. Sunflower oil was purchased from a local Mercadona store in Valencia, Spain. Glacial acetic acid (99.7% purity) and sodium thiosulfate (Na_2_S_2_O_3_, 99.5% purity) were supplied by Panreac Química S.A. (Castellar del Vallés, Spain). Finally, 1-decanol (≥99.0% purity) and potassium iodide (≥99.0% purity) were obtained from Sigma-Aldrich (Madrid, Spain).

### 2.2. Obtention of Fillers

Grape stalk samples were dried and crushed with a grinder (Model Zyklon SM 300 stainless steel, Retsch, Haan, Germany) and sieved with a 500 μm mesh. Subsequently, grape stalk (GS) powder was submitted to subcritical water extraction (SWE) at 170 °C or 180 °C for 30 min in a 5 L reactor (model 1-TAP-CE reactor, Amar Equipment Pvt. Ltd., Mumbai, India) to obtain the cellulose-enriched solid residues R170 (at 170 °C) and R180 (at 180 °C), as described by Maté et al. [22]. The samples were dried and stored in a desiccator with P_2_O_5_ prior to further use. The fillers GS, R170, and R180 were ground and sieved (63 μm) before incorporating them into the polymer matrices. The average composition and antioxidant power of the fillers are shown in Table 1, as reported by Maté et al. [22].

### 2.3. Preparation and Characterization of Biocomposites

The PBS and PHBV pellets were cold-crushed using liquid nitrogen and dried in a vacuum oven at 60 °C overnight to remove the residual water (vacuum TEM-TJP Selecta, Barcelona, Spain). An internal mixer (HAAKETM PolyLab TM QC, Thermo Fisher Scientific, Herzogenaurach, Germany) was used to obtain mixtures of PBS or PHBV with 10% wt. of GS, R170, or R180. Control films with pure PBS and PHBV were also obtained. The blending conditions were stabilized when a constant torque was reached in the torque–time curves registered by the mixer. For PBS, the blending conditions were 150 °C, 50 rpm, and 5 min, while for PHBV, these were 170 °C, 50 rpm, and 10 min.

The resulting mixtures were ground with liquid nitrogen and dried in the oven at 60 °C for one night, and films (3.4 g per film) were thermoformed in a hot plate hydraulic press (Model LP20, Labtech Engineering, Bangpoo, Thailand). The steps followed were pre-heating for 5 min at 150 °C (PBS) or 180 °C (PHBV), pressing for 4 min at 150 °C (PBS) or 180 °C (PHBV) and 100 bars, and cooling for 3 min at 60 °C. In this way, four formulations per polymer were obtained: pure polymers (PBS, PHBV), biocomposites with GS filler (PBS-GS and PHBV-GS), those with the cellulose-enriched R170 filler (PBS-R170 and PHBV-R170), and those with the cellulose-enriched R180 filler (PBS-R180 and PHBV-R180). All samples were stored at room temperature, at 0% relative humidity, or 53% relative humidity in desiccators containing P_2_O_5_ or an oversaturated solution of Mg(NO_3_)_2_.

#### 2.3.1. Microstructure, X-Ray Analysis, and FTIR of Films

To observe the morphology of the biocomposites, the samples conditioned at 0% RH were cryofractured by immersion in slush nitrogen, gold-coated (EM MED020 Leica Microsystems, Barcelona, Spain), and then examined with a field emission scanning electron microscope (ULTRA 55, Zeiss, Oxford Instruments, Oxford, UK) at a voltage of 2 kV.

The X-ray spectra of the samples were obtained with an X-ray diffractometer (AXS/D8 Advance, Bruker AXS GmbH, Karlsruhe, Germany) using Kα-Cu radiation with a λ of 1.542 Å, a voltage of 40 kV, an intensity of 40 mA, and a flow rate of 2.0 °C/min between 5 °C and 40 °C. The percentage of crystallinity in the polymer matrix was determined as the ratio of the crystalline peak area to the total area under the spectra, using the Origin software (version OriginPro 2021, OriginLab Corporation, Northampton, MA, USA).

The FTIR spectra of the films were obtained with a spectrometer (Vertex 80, Bruker AXS GmbH, Karlsruhe, Germany) equipped with an attenuated total reflectance accessory. FTIR spectra were taken in the wavelength range of 4000–500 cm^−1^, at a resolution of 6 cm^−1^, using 128 scans for each spectrum, in triplicate for each sample.

#### 2.3.2. Mechanical, Barrier, and Optical Properties of Films

The mechanical behaviour of the films was assessed by tensile tests carried out with a universal testing machine (TA.XTPlus, Stable Micro Systems, Haslemere, UK), according to the standard ASTM D882 [26]. Eight samples per formulation were cut (2.5 × 10 cm), secured in film grips at 5 cm separation, and elongated at 50 mm min^−1^ until fracture to obtain the corresponding stress vs. strain curves. From these, three mechanical parameters were obtained, namely the elastic modulus (EM), the tensile strength at break (σ), and the percentage deformation at break (ε).

The water vapour permeability (WVP) of the films was measured in triplicate following the standard ASTM E96/E96M [27], at 25 °C and a 53–100% relative humidity gradient. Round 3.5 cm diameter samples were cut and sealed on Payne permeability cups (Elcometer SPRL, Hermelle/s Argenteau, Belgium) containing 5 mL of distilled water. These were then placed into desiccators at 53% relative humidity using a Mg(NO_3_)_2_ oversaturated solution. The cups were weighed periodically up to 160 h with an analytical scale (ME36S, Sartorius, ±0.00001 g, Fisher Scientific, Hampton, NH, USA). The water vapour transmission rate, i.e., the slope of weight loss vs. time once the steady state had been reached, was used to determine the WVP expressed in cm^3^ m^−1^ s^−1^ Pa^−1^.

Oxygen permeability (OP) was measured in triplicate using Oxysense equipment (Model 8101e, Systech Illinois Ltd., Thame, UK), at 25 °C and 53% RH, following ASTM D3985-05 [28]. The measurement area per film was 50 cm^2^, and the oxygen transmission rate was measured every 15 min until the steady state was reached. Finally, OP was expressed as cm^3^·m^−1^·s^−1^·Pa^−1^.

The film thickness necessary to determine tensile and barrier properties was measured using a digital micrometer (Palmer, model COMECTA, Barcelona, Spain, accuracy of 0.001 mm) at ten random film positions. The obtained values ranged between 142 ± 13 μm for PHBV and 127 ± 13 μm for PBS with no significant effects of filler incorporation.

The optical properties of the films were measured with a spectrocolorimeter (CM-3600d, Minolta Co., Tokyo, Japan). The reflection spectra of the samples between 400 and 700 nm were measured on a white and a black background to determine the infinite reflectance (R∞), according to the Kubelka–Munk theory of multiple scattering [29]. Then, the film colour coordinates (lightness L*, redness–greenness a*, and yellowness–blueness b*) were obtained from the R_∞_ spectra, using D65 illuminant and a 10° observer. Chroma (C_ab_*) and hue (h_ab_*) were obtained from the colour coordinates a* and b*. The measurements were taken in triplicate for each sample. The colour difference between the biocomposites containing fillers and the filler-free formulation was also determined through the total colour difference parameter (ΔE*) (Equation (1)), where ΔL*, Δa*, and Δb* correspond to the differences between the colour parameters of the films.

(1) $${{\Delta E}^{*}={\left[{\left({\Delta L}^{*}\right)}^{2}+{\left({\Delta a}^{*}\right)}^{2}+{\left({\Delta b}^{*}\right)}^{2}\right]}^{1/2}}^{.}$$

The UV-vis spectra between 200 and 900 nm of the films were obtained using a UV–visible spectrophotometer (Evolution 201, Thermo Scientific, Waltham, MA, USA) operating in light transmission mode.

#### 2.3.3. Thermal Behaviour of Films

The phase transitions of the biocomposites were evaluated using a Differential Scanning Calorimetry (DSC) Stare System analyser (Mettler-Toledo GmbH, Greifensee, Switzerland), operating under a 30 mL min^−1^ nitrogen flow. The film samples (4–5 mg) were placed into aluminum pans and sealed. Each PBS sample was initially cooled down to −40 °C, heated from −40 °C to 150 °C (first heating step), cooled back to −40 °C, and finally heated (second heating step) to 150 °C, always at 10 °C min^−1^. The PHBV samples were initially heated from −40 °C to 200 °C, cooled from −200 °C to −40 °C, and heated again to 200 °C, always at 10 °C min^−1^. The crystallinity degree (Xc) of each polymer was calculated from the melting enthalpy (ΔH_m_) by using Equation (2):

(2) $$\frac{∆H_{m}-∆H_{cc}}{∆H_{m}{}^{\circ}\left(1-\omega \right)}\times 100$$

where ΔH_m_ stands for the melting enthalpy obtained from the second heating scan, ΔH°_m_ is the reported melting enthalpy of a fully crystalline polymer: 200 J g^−1^ [15] for PBS and 132 J·g^−1^ for PHBV [30]. *ω* represents the mass fraction of filler in the film formulation (0.10). ΔH_cc_ is the cold crystallization enthalpy, which is 0 for PHBV, while there is a quantitative value for PBS samples.

The thermal stability of the films was assessed in duplicate by thermogravimetric analysis (TGA) using a thermogravimetric analyzer (TGA 1 Stare System analyzer, Mettler-Toledo, Greifensee, Switzerland). The samples preconditioned at 0%RH for two weeks were weighed in alumina pans and heated from 25 to 700 °C, under nitrogen flow, at 10 °C min^−1^. The thermogravimetric curves (TGA) and their derivatives (DTGA) were analyzed to obtain the initial degradation temperature (Tonset) corresponding to 5% mass loss and the temperature at the maximum degradation rate (Tpeak).

#### 2.3.4. Antioxidant Properties of Films

To analyze the antioxidant capacity of the different films, two accelerated oxidation tests were carried out using sunflower oil in contact with the obtained materials. To determine the potential diffusion of antioxidant compounds to the oil, protecting it from oxidation, 4 mL of oil was packaged in mono-dose polyethylene (PE) bags (5.5 × 5.5 cm) containing square pieces (5 × 5 cm) of each biocomposite inside. To analyze the combined effects of oxygen and the UV light barrier of the films and the action of disused antioxidants, thermo-sealed mono-dose bags (5.5 × 11 cm) of PBS composites were filled with 4 mL sunflower oil. PHBV does not have heat sealing ability and could not be submitted to this second test. The oil bags were stored for 15 days in a chamber at 30 °C with light (T8 18W 6400 K, CH Lighting Co., Shaoxing City, China), and the oil samples were analyzed for their peroxide value and conjugated dienes and trienes, compared with the oil’s initial values [31].

### 2.4. Statistical Analyses

Data were submitted to an analysis of variance (ANOVA) at a 95% confidence level using Statgraphics Centurion XIX (version 19-X64). Differences between the formulations were determined by the Fisher test, using the least significant difference of 5% (α = 0.05).

## 3. Results and Discussion

### 3.1. Microstructural and Spectral Analysis

**Figure 1:**
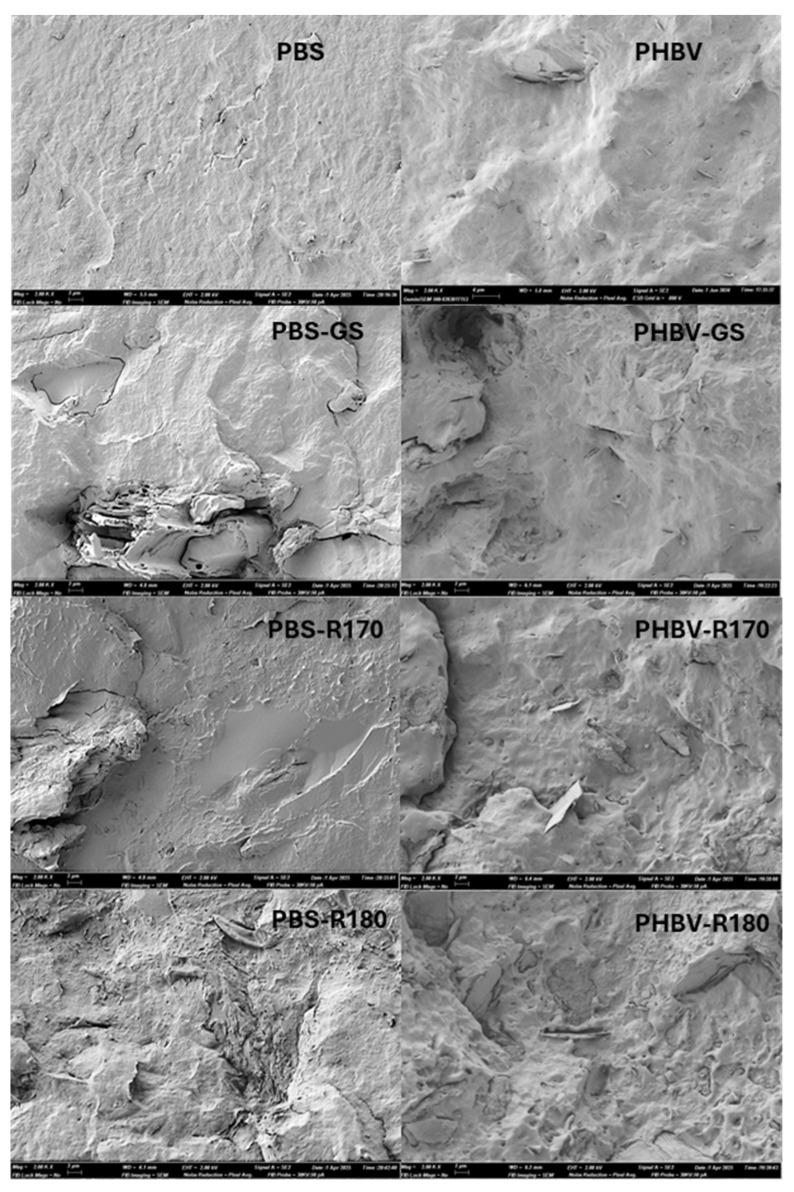
FESEM images (×2000) of cross-section of films of pure polymers (PBS, PHBV) and biocomposites with GS filler (PBS-GS and PHBV-GS), with cellulose-enriched R170 filler (PBS-R170 and PHBV-R170), and with cellulose-enriched R180 filler (PBS-R180 and PHBV-R180).

Figure 1 shows the FESEM images of the cryofracture cross-sections of pure polymers (PBS and PHBV) and their composite films. Pure polymer films showed the typical homogeneous fractures, previously described by other authors [15,30,32]. PHBV presented some particles in the polymer matrix, which probably correspond to boron nitride, normally added to commercial-grade PHBV as a nucleating agent [33]. In the composites, filler particles of different sizes could be seen as they were embedded in the polymer matrices, with different polymer–filler interactions, as affected by the particle composition (Table 1) and the polymer. During the melt blending process, different filler compounds of low molecular weight, such as phenols, could migrate into the molten polymer, establishing molecular interactions with its chains (e.g., hydrogen bonds with the carbonyl groups) and affecting the nanostructure of the film’s continuous phase. In contrast, the structural components of the particles, such as lignocellulosic fibres, remained dispersed in the polymer continuous phase. This was observed in the composites of both polymers, where different particles (or their void after cryofracture) were observed dispersed in the film’s continuous phase that also exhibited some changes in appearance. In the PBS composites, limited interfacial adhesion between the fibres and the polymer matrix could be inferred from the interfacial separation or gaps between the two phases. Interfacial gaps between the matrix and the fillers could allow for better water diffusion in the structure, as reported by Olivas et al. [15] for PBS films with rice straw cellulosic fibres. In contrast, PHBV composites showed better filler–polymer adhesion since no gaps at the interface were observed. Likewise, the particles of the R170 and R180 fillers showed a smooth appearance, being much better integrated in both the PBS and PHBV matrices. Therefore, the extraction of a part of the non-cellulosic compounds from the GS particles favoured their compatibilization with the polyester.

**Figure 2:**
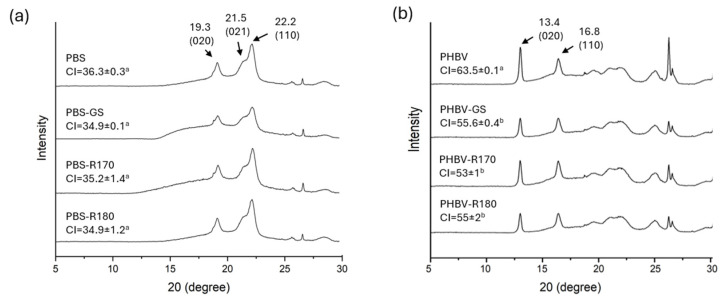
(**a**) X-ray diffraction patterns of the pure polymer PBS and biocomposites with GS filler PBS-GS with cellulose-enriched R170 filler PBS-R170, and with cellulose-enriched R180 filler PBS-R180. (**b**) X-ray diffraction patterns of the pure polymer PHBV, and biocomposites with GS filler PHBV-GS, with cellulose-enriched R170 filler PHBV-R170, and with cellulose-enriched R180 filler PHBV-R180. Different letters (a, b) indicate significant differences between samples based on the same polymer (*p* < 0.05).

The effects of fillers on polymer crystallinity were analyzed through the X-ray diffraction spectra. Figure 2 shows the X-ray diffraction patterns of the PBS-based biocomposites showing the main characteristic diffraction peaks at 2θ, 19.3°, 21.5°, and 22.2°, previously found in other studies [4,34,35], which are, respectively, assigned to the (020), (021), and (110) planes of the α-form PBS crystal [36,37]. All the samples exhibited the same diffraction peaks, which suggests that only the PBS crystalline structure is present in the composites, without changes provoked by fillers. Moreover, filler incorporation did not significantly affect the crystallinity index (CI) of the PBS biocomposites, as also observed in previous studies [16]. Despite the nucleating effect reported for some cellulosic fibres, which enhances polymer crystallization [16,38], this was not observed for GS fibres in the PBS matrix.

Figure 2 shows the X-ray diffraction patterns of the PHBV-based biocomposites. PHBV crystallizes with different structures depending on the HV content, but at a low HV percentage, the polymer crystals have the PHB structure [39], showing two sharp peaks at 2θ =13.4° and 16.8°, similar to those found in previous studies [30,40]. These are associated with the (020) and (110) diffraction planes of the α phase of the orthorhombic lattice of PHBV. Other characteristic reflection peaks appeared at 21.54°, 25.58°, and 30.06°, corresponding to the (111), (031), and (002) crystalline planes, respectively, as also reported by other authors [41]. The peak at 27° probably corresponds to the boron nitride nucleating agent [30]. No changes in the crystalline pattern occurred due to filler incorporation, but this resulted in a reduction in the intensity of the (020) peak, which may be linked to structural modifications in the crystal lattice. Moreover, the degree of crystallinity (CI) decreased with the incorporation of all fillers, revealing a certain inhibition effect of the fillers for crystallization. It could then be stated that the fillers affected the crystallization of PHBV, hindering this process and reducing the size of crystalline domains. The incorporation of lignocellulosic fillers from vine shoots also reduced crystallinity in PHBV matrices [42].

**Figure 3:**
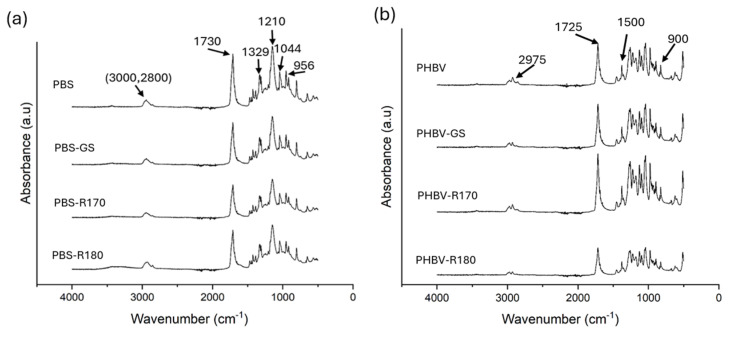
(**a**) FTIR spectra of the pure polymer PBS and biocomposites with GS filler PBS-GS with cellulose-enriched R170 filler PBS-R170, and with cellulose-enriched R180 filler PBS-R180. (**b**) FTIR spectra of the pure polymer PHBV, and biocomposites with GS filler PHBV-GS, with cellulose-enriched R170 filler PHBV-R170, and with cellulose-enriched R180 filler PHBV-R180.

The possible interactions between the polymers and the filler compounds after the melt blending process were analyzed through the FTIR spectra of the biocomposites, shown in Figure 3. For PBS films, the absorption peaks characteristic of PBS were found in all cases, coherently with previous research [43,44,45], with no noticeable effects of the fillers on the vibration bands. In the region between 3000 and 2800 cm^−1^, the asymmetric stretching of –CH- groups was found. The peak at 1730 cm^−1^ was assigned to the stretching vibrations of the carbonyl ester group [4,46,47], whereas those at 1329 and 1210 cm^−1^ corresponded to the stretching vibrations of the C-O-C group [43,44]. The peak at 1044 cm^−1^ was assigned to the stretching vibrations of the O-C-C bonds of the polymer [4,47], and finally, that at 956 cm^−1^ corresponded to the bending of the -C-OH of the terminal acid groups.

Likewise, all characteristic peaks of PHBV were observed in the FTIR spectra of the neat polymer and composites, without any noticeable effects of the filler on the registered vibration bands. Coherently with previous studies [48], these correspond to CH_3_ and CH vibrations and C-O-C and C-C stretching (peaks between 1500 and 900 cm^−1^), the characteristic peak of carbonyl stretching vibration (1725 cm^−1^), and the symmetric and asymmetric stretching of the CH_3_ group (2975 and 2937 cm^−1^). The absorption band at 2975 cm^−1^ is attributed to C-H stretching vibration in PHBV [47].

Therefore, the interactions of the polymers with the filler compounds were not markedly reflected in the FTIR spectra, probably due to the relatively low proportion of each component in the filler and the filler in the composite (10%). However, as expected, the intensity of the bands was attenuated by filler incorporation.

### 3.2. Mechanical, Barrier, and Optical Properties

Tensile tests were carried out on the films to assess the filler effect on the mechanical yield of composites. Table 2 shows the elastic modulus (EM) and tensile and deformation at break of the different films. The tensile behaviour of pure PBS films was coherent with that reported in previous studies, typical of rigid materials with low plastic deformation and a relatively low Young’s modulus (300–500 MPa), depending on its degree of crystallinity [4,49]. In contrast, pure PHBV exhibited a high elastic modulus in the range of 2–3 GPa which, in addition to the rest of its tensile properties, was in the range previously reported [50,51]. The high rigidity and brittleness of PHBV are related to its high crystallinity and large spherulites [41]. Controlling the crystallization in the PHBV matrix in different ways, such as filler incorporation, may be a useful strategy to modulate its mechanical properties.

The tensile properties of the films were affected by the addition of fillers. As compared to pure PBS films, PBS composites were stiffer while also presenting decreased resistance to break and elongation. These changes in the mechanical parameters are the typical response to the incorporation of rigid fillers into a softer polymer matrix [15,52]. Since the elastic modulus is evaluated at low deformations, where particle–matrix separation phenomena generally do not occur, it is only moderately affected by particle–polymer interactions [16]. Therefore, this increase is mainly explained by the intrinsic mechanical properties of the fillers, which are highly dependent on their composition. The most remarkable increase in the modulus was observed in the samples PBS-R170 and PBS-R180, which are richer in cellulose (Table 1), exhibiting more rigidity. As generally observed, filler incorporation resulted in reduced extensibility for PBS biocomposites, due to the chain mobility restrictions imposed by the filler and the discontinuities introduced into the polymer matrix.

A different effect of the fillers was observed for the PHBV composites, where a reduction in the elastic modulus was observed in every case. In previous studies, an increase in the rigidity of PHBV-based films was observed due to the incorporation of plant fibres, namely sisal [51], bamboo [53], flax, hemp and wood [54], or jute and abaca [50]. In contrast, GS fillers caused a remarkable reduction in the stiffness in PHBV films, without a significant effect of their composition. This could be mainly attributed to the partial hydrolysis of the polymer during melt blending with the fillers due to the high sensitivity of PHBV to water hydrolysis [55]. The highly hydrophilic nature of the fillers (rich in water extractable compounds, Table 1) is associated with a significant fraction of bound water that triggers the chain scission process during the melt blending process. The lower crystallinity of the composites deduced from the XRD analysis also contributes to the reduction in rigidity.

The film barrier capacity, both against oxygen and water vapour, is important to ensure material adequacy for food packaging. The incorporation of fillers had a relevant impact on oxygen and water vapour film barrier properties, with some differences between fillers, as shown in Table 2. The OP of pure PBS films was similar to previously reported values [15], exhibiting a medium-to-low barrier to oxygen [56]. Likewise, the lower OP value obtained for pure PHBV was in the range reported by other authors [57]. The oxygen barrier performance of the composites of both polymers was significantly improved (*p* < 0.05) via filler incorporation (by about 20 and 35%, respectively, for PBS and PHBV), without significant differences caused by the type of filler. This improvement can be attributed to different factors. First, the fillers promote an increase in the tortuosity factor for mass transfer due to their lower affinity for oxygen, which reduces permeability by affecting the path of the permeant through the polymer structure [58]. In addition, the fillers exhibited antioxidant activity, as shown in Table 1 [22], which may also contribute to the oxygen barrier effect through an oxygen scavenging effect [59]. The promotion of the oxygen barrier in PBS and PHBV films by incorporating lignocellulosic fillers has also been observed by other authors [15,60].

The WVP values of pure PBS and PHBV films were similar to those previously reported [15,30], with the PHBV films exhibiting higher water barrier capacity than PBS. The incorporation of fillers in PBS composites markedly (near 100%) increased the WVP values (*p* < 0.05), due to the hydrophilic nature of the particles that promoted water affinity, and thus, this did not contribute to an effective tortuosity factor for the transfer of water molecules. Likewise, the lack of good interfacial adhesion between fillers and the polymer could promote water transfer though the interfacial gaps, as mentioned in the section of this paper discussing the microstructure. The potential partial hydrolysis of the polymer, promoted by the filler bound water, may also enhance the WVP values. Olivas-Alonso et al. [15] also observed a similar effect in the WVP and OP values of PBS films by incorporating cellulose fibres from rice straw. The compositional changes provoked by SWE in the fillers (Table 1) did not result in significant WVP differences among the composites.

In contrast, filler incorporation into the PHBV matrix resulted in a slight WVP decrease (16–30%), which was more marked for the cellulose-enriched fillers R170 and R180. This could be, in part, attributed to the better compatibility between the two phases observed in the FESEM images, where R170 and R180 particles were better integrated in the matrix. Other authors [14] reported an increase in the WVP of PHBV composites with lignocellulosic fibres. Nevertheless, the interactions of the PHBV chains with the other molecular compounds, such as phenols, released from the fillers could mitigate this effect in the PHBV matrix. Other authors [30] found a small decrease in the WVP of the PHBV matrix when incorporating phenolic acids.

**Figure 4:**
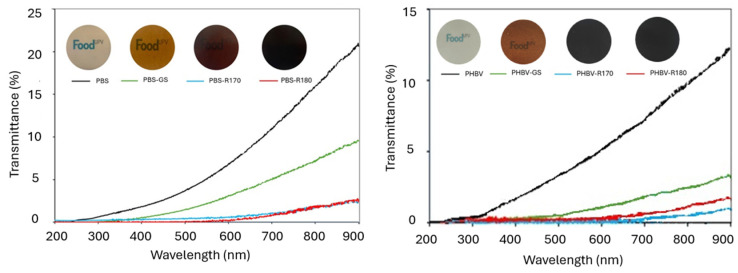
Images and transmittance UV-vis spectra of pure polymers (PBS, PHBV) and biocomposites with GS filler (PBS-GS and PHBV-GS), with cellulose-enriched R170 filler (PBS-R170 and PHBV-R170), and with cellulose-enriched R180 filler (PBS-R180 and PHBV-R180).

The incorporation of the fillers had a great impact on the interactions of the films with light, contributing not only to their appearance but also to their light barrier capacity. Figure 4 shows the UV-vis transmittance spectra of the different films along with film images, and Table 3 shows the colour coordinates (lightness: L*; chrome: C_ab_*; hue: hab*) of the different films, along with the colour difference with respect to the corresponding pure polymer films. PHBV films were opaquer than PBS films, with lower transmittance values throughout the entire wavelength range, also exhibiting different colour characteristics. Pure PHBV films were darker (lower L*), more yellowish (higher h_ab_*), and more saturated in colour (higher C_ab_*) than pure PBS films. Likewise, a greater reduction in transmittance was produced in PHBV films when fillers were incorporated. This suggests that there are different interactions of the filler compounds with the polymer chains affecting light transmission, which could indicate a greater release of compounds from the filler to the polymer matrix during melt blending.

Incorporating the fillers decreased the films’ transmittance in the complete wavelength range (200 to 900 nm), mainly in the UV region where the GS phenolic constituents were absorbed. Samples with R170 and R180 showed very low transmittance values, which implies a high light barrier capacity of these composites and makes them appropriate for protecting food products sensitive to light-induced oxidative processes. The lower transmittance of composites with both the R170 and R180 fillers could be attributed to the formation of brown compounds during SWE at high temperature, in line with the occurrence of Maillard reactions and sugar caramelization, as described in previous studies [61,62].

The incorporation of the fillers, especially R170 and R180, resulted in a remarkable decrease in film lightness and changes in the chromatic parameters, which could also be noticed in the film images (Figure 4). All composites became redder than the corresponding film of the net polymer, especially those containing the R170 and R180 fillers, which also reduced the film colour saturation more and promoted the colour difference with respect to the pure polymer films.

### 3.3. Thermal Behaviour of Films

**Figure 5:**
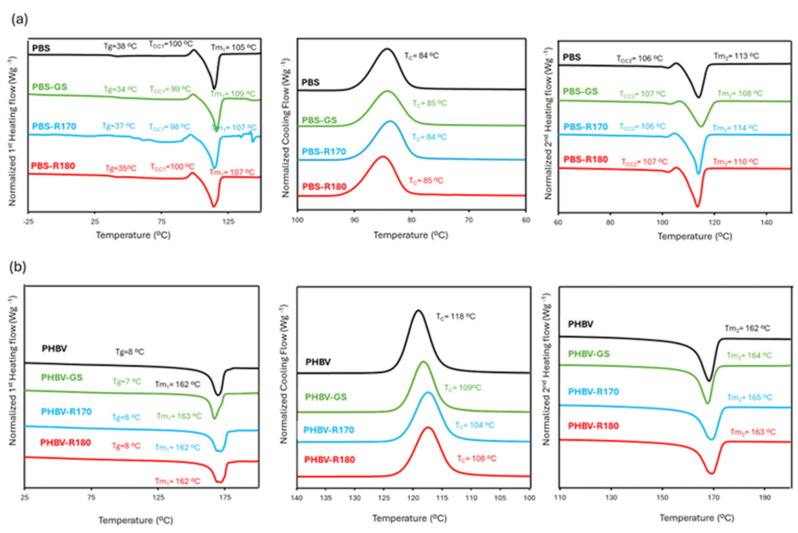
(**a**) DSC thermograms of pure polymers PBS and biocomposites with GS filler PBS-GS, with cellulose-enriched R170 filler PBS-R170, and with cellulose-enriched R180 filler PBS-R180 and. (**b**) DSC thermograms of pure polymers PHBV and biocomposites with GS filler PHBV-GS, with cellulose-enriched R170 filler PHBV-R170, and with cellulose-enriched R180 filler PHBV-R180.

DSC studies of the pure polymers and their composites were carried out in order to understand the effects of the fillers on the polymer phase transitions. Figure 5 shows the thermograms of the different film samples obtained in the first heating, cooling, and the second heating steps, reflecting the temperatures of the detected thermal transitions. Likewise, the values of melting enthalpy and the calculated crystallinity from the heating steps are shown in Table 4. For PBS, cold crystallization was observed, and the corresponding enthalpy values were deduced from the estimated ΔH_m_ values to determine the crystallinity percentage in the polymer.

In the first heating step of PBS films (Figure 5), a second-order transition was observed at about 35–38 °C, which suggests the presence of the glass transition of PBS other than the main one reported between −25 °C and −45 °C [15,44,45], which was not detected in the present study. This second high-temperature glass transition was also reported by Olivas-Alonso et al. [15] and attributed to the amorphous phase near the crystalline domains with more restricted molecular mobility due to its connection to the crystal surface [15]. Endothermic peaks corresponding to the melting of PBS were observed at 105–110 °C, coherently with previous studies [45], which were preceded by a small exothermic peak associated with polymer cold crystallization at about 106 °C, with enthalpy values of 1.9–2.7 J/g of the polymer. This reveals that there is a PBS recrystallization or rearrangement from the metastable lamellae with increasing temperature [5,9,63]. In agreement with previous studies [16], the incorporation of fillers into PBS matrices did not have a significant effect on the temperatures or enthalpies of cold crystallization and melting (Figure 5, Table 4). Likewise, the crystallinity index estimated from the first heating step was similar for the different composites (33–34%), as found by XRD analysis. As previously reported [43,44,45], the crystallization peak of pure PBS occurs at about 85 °C (Figure 5b), without a significant influence of the fillers. So, no nucleating or antinucleating effect could be deduced for the fillers, coherently with that deduced from the XR diffraction spectra.

The thermograms of pure PHBV films showed a glass transition at about 8 °C, as previously reported [30]. Likewise, the crystallization and melting temperatures (120 °C and 162 °C, respectively) were in the previously reported range [30,53,64]. Incorporating the fillers into the PHBV matrix did not significantly affect (*p* > 0.05) the glass transition and melting temperatures, but it did reduce the crystallization temperature, indicating that a higher degree of supercooling was necessary to promote PHBV crystallization when fillers were present. This has been observed in PHBV blends [55] when no component acts as a nucleating agent, due to the blending effect. Moreover, the melting enthalpies of composites were lower than those of net PHBV. Therefore, fillers inhibited the crystallization of PHBV, regardless of their composition, as also deduced from the XRD analysis. This behaviour also suggests a higher release of low molecular compounds from the fillers to the PHBV matrix, interacting with the polymer chains and affecting their crystallization.

**Figure 6:**
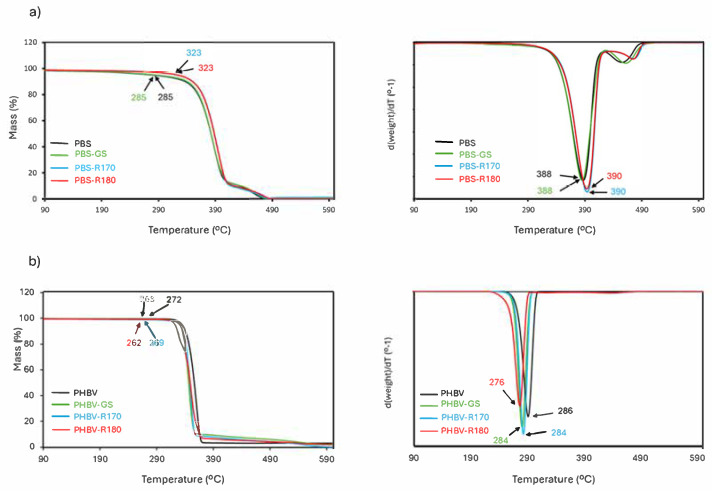
TGA (**a**) and DTGA curves (**b**) of pure polymers (PBS, PHBV) and biocomposites with GS filler (PBS-GS and PHBV-GS), with cellulose-enriched R170 filler (PBS-R170 and PHBV-R170), and with cellulose-enriched R180 filler (PBS-R180 and PHBV-R180). Onset and peak temperatures are marked in plots.

The thermal stability of the films was studied by thermogravimetric analysis, as reflected in TGA and DTGA curves (Figure 6). PBS materials decomposed in two steps, with the first one, starting at about 300 °C, having a maximum degradation rate at about 390 °C (T_max_), which is related to the thermal degradation of the polymer [36,43,44]. The second mass loss step at a higher temperature was attributed to the decomposition of organic mass produced in the first step [15,44]. On the other hand, one-step PHBV thermal degradation occurred at a lower temperature, close to 300 °C, coherently with previous studies [33,42,48].

The fillers had differing effects on the thermal stability of the polymers. For PBS composites, the GS filler did not significantly affect the polymer degradation pattern, whereas cellulose-enriched fillers (R170 and R180) slightly enhanced the thermal stability of the polymer, raising the temperature at onset (T_onset_) and the maximum degradation rate (T_peak_ in DTGA curve). This effect can be attributed to the higher cellulose content of these fillers, with high thermal resistance. In contrast, PHBV composites exhibited a reduction in thermal stability (lower values of T_onset_ and T_peak_), which can be attributed to the partial hydrolysis of the polymer chains during melt blending, promoted by the bound water in fillers, as also deduced from the changes in the tensile parameters of composites. Moreover, the reduction in polymer crystallinity caused by filler incorporation may have an additional effect on the reduction in the thermal stability of PHBV.

### 3.4. Antioxidant Properties

The antioxidant properties of the films were evaluated through their ability to control the oxidation of sunflower oil in contact with the materials. For simple contact, in oil packaged in PE bags containing film pieces inside, Table 5 shows the values of the oxidation parameters (peroxide index and conjugated dienes and trienes) after 15 exposure days, in comparison with the oil’s initial values. A significant increase in all parameters was observed in the control sample (oil in PE bags), validating the progress of oxidation under the test conditions. For PBS films, no significant differences between oxidation parameters with respect to the control sample were observed, which suggests that no significant migration of antioxidant compounds occurred from the composites to oil to protect it from oxidation. In contrast, a significant reduction in the oxidation parameters, with respect to the control sample, occurred for PHBV composites with the different antioxidant fillers. This suggests the effective migration of the antioxidant compounds from the PHBV composites, contributing to reduce the oil oxidation rate. Nevertheless, no significant differences in oxidation protection could be deduced for the different fillers despite their different DPPH scavenging capacities (Table 1). This suggests that the migrated compounds are specific to GS and were not affected by extraction in subcritical water.

**Figure 7:**
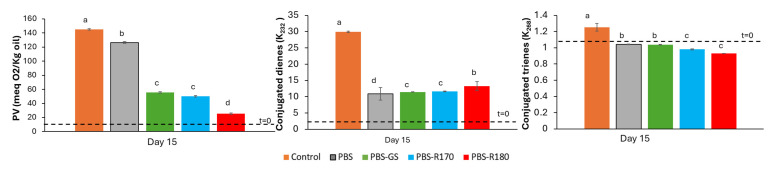
Peroxide value (PV) and conjugated dienes and trienes of sunflower oil samples after 15 days storage in mono-dose bags of pure polymer (PBS) and biocomposite with GS filler (PBS-GS), with cellulose-enriched R170 filler (PBS-R170), and with cellulose-enriched R180 filler (PBS-R180). Control sample: unpackaged oil. Initial values in oil are marked with dashed line. Different letters (a, b, c, d) indicate significant differences among oil samples (*p* < 0.05).

For oil packaged in bags of PBS materials, Figure 7 shows the values of the oxidation parameters after 15 exposure days, compared with the initial values and those of the control sample (unpackaged oil). As mainly revealed by the values of the peroxide index (PV), PBS composites were remarkably more effective than pure PBS films for sunflower oil preservation under the accelerated oxidation conditions tested. This protective effect can be attributed to the improved barrier properties to both oxygen and UV light, as observed by other authors [23], since no significant migration of antioxidants to the oil could be deduced from the previous test.

## 4. Conclusions

Antioxidant biocomposite films of PBS or PHBV with lignocellulosic particles of GS with different compositions could be successfully obtained by melt blending and compression moulding. The fibres were better integrated in the PHBV matrix, while other molecular compounds from the fillers seemed to be released into the polymer matrix, which allows for their antioxidant action in contact with oil. Fillers promoted the stiffness of PBS films, reducing their resistance to break and extensibility, without significant changes in polymer crystallinity or thermal stability. However, this reduced the crystallinity and thermal stability of PHBV films, decreasing their rigidity, without remarkable changes in the resistance and deformation at break. All fillers promoted the oxygen barrier capacity of the PHBV and PBS matrices while also providing them with a UV light blocking effect. These barriers enhanced the ability of the films to preserve sunflower oil against oxidation, while in PHBV composites, the migration of antioxidant compounds was also detected. Despite the composition of fillers affecting some properties of the composites, such as the stiffness in PBS films or the oxygen barrier in PHBV films, from a practical point of view, the use of an untreated GS filler is recommended to avoid the cost of additional processing. Further studies are required to validate these composites as antioxidant packaging materials in different oxidation-prone foods.

## Figures and Tables

**Table 1:** Average content (g/100 g sample) of water extractives, structural compounds, and ashes; total phenolic content (TPC); and DPPH scavenging capacity (EC50) of different fillers (grape stalk (GS) and the SWE solid residues obtained at 170 °C (R170) and 180 °C (R180)). Data are as reported by Mate et al. [22].

Sample	Water Extractives (g/100 g)	Cellulose (g/100 g)	Hemicellulose (g/100 g)	Lignin (g/100 g)	Ashes (g/100 g)	TPC (g GAE/100 g)	EC 50 (mg/mg DPPH)
GS	52.2 ± 0.0 ^a^	22 ± 4 ^a^	7.0 ± 4 ^a^	14.7 ± 1.3 ^a^	7.6 ± 0.1 ^a^	9.3 ± 0.6 ^c^	0.64 ± 0.07 ^a^
R170	25.9 ± 0.0 ^b^	32 ± 4 ^b^	1.6 ± 0.1 ^b^	41.3 ± 1.6 ^b^	2.9 ± 0.1 ^b^	17.8 ± 0.3 ^b^	0.38 ± 0.05 ^b^
R180	21.2 ± 0.1 ^b^	34 ± 1.8 ^b^	0.4 ± 0.1 ^b^	40.0 ± 0.2 ^b^	2.9 ± 0.1 ^b^	19.7 ± 0.3 ^a^	0.38 ± 0.01 ^b^

Different letters (a, b, c) indicate significant differences between samples (*p* < 0.05).

**Table 2:** Mechanical and barrier properties of pure polymers (PBS, PHBV) and biocomposites with GS filler (PBS-GS and PHBV-GS), with cellulose-enriched R170 filler (PBS-R170 and PHBV-R170), and with cellulose-enriched R180 filler (PBS-R180 and PHBV-R180).

Sample	EM (MPa)	σ (MPa)	ε (%)	OP × 10^14^ (cm^3^ m^−1^ s^−1^ Pa^−1^)	WVP × 10^12^ (g m^−1^ s^−1^ Pa^−1^)
PBS	448 ± 21 ^c^	40.8 ± 1.7 ^a^	9.0 ± 0.6 ^a^	86.0 ± 3.0 ^a^	18.0 ± 2.0 ^b^
PBS-GS	504 ± 49 ^b^	31.3 ± 1.9 ^b^	6.1 ± 0.9 ^b^	73.2 ± 0.3 ^b^	36.0 ± 5.0 ^a^
PBS-R170	634 ± 23 ^a^	27.0 ± 2.0 ^c^	4.5 ± 0.6 ^c^	74.0 ± 2.0 ^b^	39.0 ± 6.0 ^a^
PBS-R180	664 ± 55 ^a^	29.5 ± 1.9 ^bc^	4.6 ± 0.6 ^c^	65.4 ± 1.7 ^c^	38.0 ± 8.0 ^a^
PHBV	2500 ± 180 ^a^	33.0 ± 2.0 ^a^	1.7 ± 0.2 ^b^	39.0 ± 2.0 ^a^	6.0 ± 0.2 ^a^
PHBV-GS	1100 ± 120 ^b^	27.0 ± 3.0 ^c^	1.8 ± 0.1 ^b^	26.0 ± 2.0 ^b^	5.1 ± 1.2 ^a^
PHBV-R170	1200 ± 200 ^b^	32.0 ± 4.0 ^ab^	2.2 ± 0.1 ^a^	26.0 ± 6.0 ^b^	4.5 ± 0.4 ^b^
PHBV-R180	1200 ± 120 ^b^	30.0 ± 3.0 ^bc^	1.9 ± 0.1 ^ab^	23.0 ± 5.0 ^b^	4.2 ± 0.2 ^b^

Different letters (a, b, c) indicate significant differences between samples based on the same polymer (*p* < 0.05).

**Table 3:** Optical properties of pure polymer films (PBS, PHBV) and biocomposites with GS filler (PBS-GS and PHBV-GS), with cellulose-enriched R170 filler (PBS-R170 and PHBV-R170), and with cellulose-enriched R180 filler (PBS-R180 and PHBV-R180).

Sample	L*	C_ab_*	h_ab_*	∆E*
PBS	93 ± 0 ^a^	5.40 ± 0.10 ^b^	69.3 ± 0.3 ^a^	-
PBS-GS	41.0 ± 1.0 ^b^	21.5 ± 0.2 ^a^	57.9 ± 1.1 ^b^	58.3 ± 0.8 ^b^
PBS-R170	23.9 ± 0.2 ^c^	1.80 ± 0.10 ^c^	31.0 ± 2.0 ^c^	69.4 ± 0.2 ^a^
PBS-R180	24.0 ± 0.0 ^c^	1.20 ± 0.10 ^d^	30.0 ± 4.0 ^c^	69.7 ± 0.0 ^a^
PHBV	75.0 ± 0.1 ^a^	18.6 ± 0.2 ^a^	80.5 ± 0.0 ^a^	-
PHBV-GS	37.1 ± 0.0 ^b^	13.4 ± 0.1 ^b^	51.1 ± 0.1 ^b^	39.1 ± 0.1 ^c^
PHBV-R170	27.4 ± 0.0 ^c^	2.3 ± 0.1 ^c^	41.8 ± 0.1 ^c^	50.2 ± 0.1 ^b^
PHBV-R180	27.7 ± 0.0 ^d^	2.5 ± 0.0 ^d^	41.7 ± 0.1 ^c^	50.6 ± 0.0 ^a^

Different letters (a, b, c, d) indicate significant differences between films based on the same polymer (*p* < 0.05).

**Table 4:** Melting enthalpy and crystallization degree of pure polymers (PBS, PHBV) and biocomposites with GS filler (PBS-GS and PHBV-GS), with cellulose-enriched R170 filler (PBS-R170 and PHBV-R170), and with cellulose-enriched R180 filler (PBS-R180 and PHBV-R180).

	1st Heating		2nd Heating	
Sample	∆H_m_ (J/g Polymer)	Xc (%)	∆H_m_ (J/g Polymer)	Xc (%)
PBS	67.0 ± 3.0 ^a^	33.2 ± 1.3 ^a^	60.0 ± 6.0 ^a^	29.7 ± 2.8 ^a^
PBS-GS	66.6 ± 0.6 ^a^	32.7 ± 0.2 ^a^	57.8 ± 0.5 ^a^	28.6 ± 0.5 ^a^
PBS-R170	69.6 ± 1.14 ^a^	34.5 ± 0.6 ^a^	58.0 ± 3.0 ^a^	28.7 ± 0.2 ^a^
PBS-R180	69.0 ± 6.0 ^a^	34.0 ± 3.0 ^a^	57.7 ± 1.6 ^a^	28.6 ± 0.4 ^a^
PHBV	86.9 ± 0.4 ^a^	65.8 ± 0.3 ^a^	86.0 ± 2.0 ^a^	65.0 ± 2.0 ^a^
PHBV-GS	65.0 ± 2.0 ^b^	49.0 ± 2.0 ^b^	67.0 ± 3.0 ^b^	51.0 ± 2.0 ^b^
PHBV-R170	69.0 ± 8.0 ^b^	52.2 ± 0.3 ^b^	70.3 ± 1.1 ^b^	53.2 ± 0.8 ^b^
PHBV-R180	66.0 ± 1.7 ^b^	50.0 ± 1.3 ^b^	71.6 ± 0.3 ^b^	54.5 ± 0.3 ^b^

Different letters (a, b) indicate significant differences between films based on the same polymer (*p* < 0.05).

**Table 5:** Peroxide value (PV), conjugated dienes, and conjugated trienes of sunflower oil at initial time and after storage (15 days) in PE bags containing film pieces of pure polymers (PBS, PHBV) and biocomposites with GS filler (PBS-GS and PHBV-GS), with cellulose-enriched R170 filler (PBS-R170 and PHBV-R170), and with cellulose-enriched R180 filler (PBS-R180 and PHBV-R180). Control: PE bags without film sample inside.

Samples	Time (Days)	PV (meq O_2_/Kg Oil)	Conjugated Dienes (K_232_)	Conjugated Trienes (K_268_)
	0	5.36 ± 0.04	3.34 ± 0.01	1.07 ± 0.01
Control	15	145.0 ± 12.0 ^a^	29.93 ± 0.2 ^c^	1.25 ± 0.05 ^ab^
PBS		124.4 ± 11.2 ^a^	29.2 ± 1.2 ^b^	1.47 ± 0.08 ^ab^
PBS-GS		119.0 ± 20.0 ^a^	29.8 ± 0.8 ^b^	1.36 ± 0.03 ^ab^
PBS-R170		122.6 ± 11.3 ^a^	29.3 ± 1.7 ^ab^	1.25 ± 0.03 ^ab^
PBS-R180		124.0 ± 3.0 ^a^	29.3 ± 0.2 ^b^	1.40 ± 0.20 ^b^
PHBV		128.0 ± 18.0 ^a^	29.4 ±0.7 ^ab^	1.20 ± 0.16 ^ab^
PHBV-GS		84.0 ± 22.0 ^b^	25.6 ± 1.4 ^a^	1.70 ± 0.40 ^b^
PHBV-R170		79.6 ± 0.2 ^b^	26.0 ± 2.0 ^ab^	1.18 ± 0.05 ^ab^
PHBV-R180		86.0 ± 9.0 ^b^	28.2 ± 1.5 ^ab^	1.13 ± 0.05 ^a^

Different superscript letters indicate significant differences among samples (*p* < 0.05).

## Data Availability

The data used to support the findings of this study can be made available by the corresponding author upon request.
